# Utility of the Atopy Patch Test in the Diagnosis of Allergic Rhinitis

**Published:** 2016-05

**Authors:** Nicola Fuiano, Cristoforo Incorvaia

**Affiliations:** 1 *Pediatric Allergy Service, San Severo, Italy.*; 2 *Allergy/Pulmonary rehabilitation, ICP Hospital, Milan, Italy.*

**Keywords:** Allergic rhinitis, Allergens, Atopy patch test, Diagnostic tests, Sensitization

## Abstract

**Introduction:**

The diagnostic work-up of allergic rhinitis (AR) is first and foremost based on the combination of clinical history data and results of skin prick tests (SPT). Other tests, including specific IgE measurement, nasal challenge, and, as a third option, component resolved diagnosis or basophil activation test, may be useful when the diagnosis is difficult because of polysensitization or when negative results of SPT are observed despite a suggestive history for allergy. The atopy patch test (APT) that assesses the type 4 delayed hypersensitivity allergy is currently not sufficiently used. The data obtained in recent studies on the diagnostic utility of the APT in patients with AR was reviewed.

**Data Sources:**

Review of the literature.

**Conclusion:**

The data from available studies show that the APT is frequently positive in patients with AR, especially when there is a positive history for atopic dermatitis. Very often, APT is the only positive test and therefore performing only SPT or in vitro IgE measurement may lead to an erroneous diagnosis of nonallergic rhinitis. Recent data suggest a role for APT not only for diagnosis but also in epidemiological investigation on respiratory allergy.

## Introduction

Allergic rhinitis (AR) is a very common chronic disease (1), that often begins in childhood and adolescence and then persists for a long time (2). In adults, a recent survey on 3383 patients showed that the moderate to severe forms of AR prevail over the mild forms (3). This results in a significant impact on the health and quality of life of patients in addition to a relevant social burden (4,5), that is further increased by the occurrence of frequent comorbidities such as conjunctivitis and asthma(6). 

In the US the annual direct costs of AR were estimated to be from $2 to $5 billion, while indirect costs range from $5, 5 to $9,7 billion (4). 

An accurate diagnosis is required to properly manage patients with AR and to reduce such great costs. The first step is to differ allergic from nonallergic rhinitis by performing allergy tests. The most common test, which is recommended as a first diagnostic step according to the guidelines, is the skin prick test (SPT) with allergen extracts, followed by in vitro measurement of allergen-specific IgE antibodies and a nasal challenge test (7-9). Additionally, it is also possible to use component resolved diagnosis (CRD) as third level investigation to detect the IgE to single allergen components in addition to the basophil activation test (BAT) (10), which tests the allergen reactivity of basophils (11). All these tests assess the type 1, IgE-mediated allergic reaction, but recent studies showed that the type 4, T-cell-mediated mechanism, may often be responsible for allergic rhinitis. The atopy patch test (APT) is aimed at detecting the T-cell-mediated reactions underlying allergic diseases. Since the studies published so far on APT are not randomized controlled trials, a systematic review of literature is not feasible. 

Here we review and analyze the available data on the role of the APT in diagnosing a T-cell-mediated allergic sensitization in patients with respiratory allergy.

## The Common Diagnostic Work-up for AR

The diagnosis of AR if often reached by combining history data, particularly during the period of the year when the symptoms occurred, and the results of SPT. This is easier to obtain for pollen-induced rhinitis, because the periods of pollen production for different botanical species are well known. For example, in a patient with typical symptoms, such as sneezing, runny nose, nasal itching, and nasal obstruction, occurring in the months of May and June and positive SPT to grass pollen extracts, no other diagnostic test is needed. Problems arise when multiple positive results to SPT occur, because they define the patient as polysensitized and require distinguishing the allergen linked to simple sensitization from those eliciting clinical allergy (12,13). Another issue is in the case where negative tests are administered, which is quite common but may actually be a false negative result. In both issues, additional testing is needed. In patients with apparent polysensitization, the diagnostic tool, which is currently the most feasible tool to identify the true causative allergen, is CRD. Indeed, the data provided by detecting specific IgE to the single molecular allergen components may clearly improve the precision of the diagnosis (14,15). When using such methods, it is preferable to test patients’ serum specifically with the suspected allergens rather than testing large panels of molecules, such as in the microarray technique, because the incidental detection of IgE to unexpected allergens, such as food or venom allergens, places the physician in a dilemma of how to manage the issue (16). 

On the other hand, false negative results of common IgE tests also prevent correct diagnosis. Recent studies highlighted that an exclusively local production of IgE in the nasal mucosa is not as rare as believed. In fact, Rondon et al. found that in a population of patients with suspected AR, around 25% had a negative result to SPT and in vitro IgE tests but had a positive result to nasal challenge, which revealed the presence of IgE in the nose. They named this clinical entity “local allergic rhinitis” (LAR) and demonstrated that in patients with seasonal rhinitis (17), that was labeled as idiopathic because of negative tests, a Th2-IgE-mediated nasal inflammation was present, as demonstrated by a nasal leukocyte-lymphocyte phenotype (CD45, CD33, CD16, CD3, CD4 and CD8), eosinophil-cationic-protein, and total and specific-IgE to grass and olive pollen in nasal lavage (18). LAR can also be diagnosed by directly detecting IgE in the nasal mucosa with a method based on incubating a solid-phase coupled allergen with the IgE antibody onto the mucosal surface by a specific applicator (19). Interestingly, by performing such a test in 192 patients with positive SPT to aeroallergens (111 symptomatic and 81 asymptomatic) the nasal IgE test was positive in 77.5% of symptomatic patients and in only 13.6% of asymptomatic patients (P<0001), suggesting that the absence of specific IgE in the nasal mucosa might explain the absence of symptoms in subjects with allergic sensitization assessed by SPT (20). These findings suggest the importance of undergoing a proper search of the allergic causes of rhinitis, principally of house dust mites, in patients with negative results to usual allergy tests (21). In particular, investigating the type 4 cell-mediated hypersensitivity mechanism by the APT should be carefully considered in this clinical context.

## The Emerging role of the APT

The APT was introduced by Ring et al. in 1989 to evaluate the role of aeroallergens in atopic dermatitis (AD) (22). In the following years, a number of studies provided convincing demonstration on the ability of the APT to reproduce the pathophysiology of AD. A skin biopsy study showed that 24 hours after the application of APT with dust mites a Th2 oriented cytokine pattern was detected, but after 48 hours a shift to a Th1 pattern was observed, similarly to what actually occurs in the typical skin lesions of AD (23). Wistokat-Wülfing et al. compared the APT results with those from allergen-specific IgE, specific lymphocyte proliferation, and the expression of 'activation' markers on peripheral blood T-cells after in vitro stimulation with dust mites, cat or grass pollen allergens. 48% of patients sensitized to aeroallergens developed APT reactions to the corresponding allergen, which was significantly associated with allergen specific lymphocyte proliferation (24). In addition, APT application to the skin of subjects with AD was followed by an influx of inflammatory dendritic epidermal cells (25). Importantly, in patients with a diagnosis of intrinsic AD, because of negative IgE tests, a positive APT for dust mites was frequently observed (26). This concept was reinforced in a European multicenter study on patients with AD, which reported a frequency of positive APT reactions to dust mites in 39% of patients and a positive APT without positive SPT or sIgE for the respective allergen in 17% of patients (27). 

An advance in the clinical role of the APT was achieved by studying its positive results in subjects with respiratory allergy. Guler et al. reported that in children with rhinitis or asthma, with positive results to SPT with Dermatophagoides pteronyssinus and no history of AD, 25% of subjects had a positive APT. This lead the authors to conclude that patch testing with mites may partly identify sensitive children with respiratory allergy and thus that positive APT results may imply that delayed hypersensitivity reactions play a role in children with asthma and rhinitis allergies to mites (28). Indeed, subsequent studies showed that a positive history for AD is a significant risk factor to develop a T-cell mediated hypersensitivity also inducing rhinitis and asthma. In a study published in 2008, 297 children with a positive history of AD were divided into 3 groups: current AD, current AD with respiratory symptoms, and past AD with respiratory symptoms, with 49 patients with rhinitis or asthma but with no history of AD serving as control group. Significantly more frequent positive results were found for APT in patients with AD (P<001) and for SPT in the control group compared with the other groups (P<003). Multivariate analysis showed high odds ratios (OR) concerning the probability of having AD in patients with positive APT results (OR, 21.9) and of having a positive APT result in patients with AEDS (OR, 17.4). Such a probability was even seen in patients with current AD and respiratory disease (OR, 21.9) and in patients with past AEDS and respiratory disease (OR, 22.8) (29). This suggested that the mechanisms underlying the skin response to the APT are related to the pathogenesis of AD, which is highly complex and increasingly investigated (30). The pivotal roles are sustained by the skin barrier dysfunction, which allows the passage of aeroallergens, particularly mite allergens, the allergen handling by dendritic cells and their interaction with lymphocytes, which influences the onset of a T-cell response in the skin. This response is initially of the TH2 type but later switches to a TH1 type and to a systemic TH2 response, inducing the isotype switching to IgE synthesis and the involvement of eosinophils.

A further study on 399 patients expanded the evaluation to in vitro measurement of specific IgE. The same subgroup division as in the previous study was done (current AD, current AD plus respiratory symptoms, and past AD with respiratory symptoms, with 65 patients with rhinitis or asthma but without past or current AD serving as a control group). The APT was significantly more frequently positive in the groups with current AD or past AD, compared to the control group, while SPT and specific IgE in serum were significantly more frequently positive in the control group (31). These observations stimulated a reappraisal of the role of APT in the diagnosis of rhinitis and asthma, especially in patients with a positive history (past or current) for AD (32).

The latest developments included the evaluation of the diagnostic performance of APT and its application in epidemiological studies. In a study on 468 children and adolescents with respiratory symptoms with or without a history of AD and 53 healthy controls, all subjects underwent SPT, APT, and specific IgE measurement to inhalant allergens (including grass pollen, cypress pollen, Compositae pollen, Alternaria, cat epithelium, and Dermatophagoides). The specificity and the positive and negative predictive values were calculated for each test. APT showed a specificity of 98.2%, a positive predictive value of 99.2%, and a negative predictive value of 16.1%, compared with 94.3%, 96.8%, and 11% for SPT and 96.2%, 97.9%, and 11.5% for specific IgE in serum, respectively (33). The authors concluded that in young patients sensitized to inhalant allergens with AD in addition to respiratory symptoms, the APT has a superior diagnostic performance compared with SPT and in vitro IgE measurement. This support the use of APT, which had not recently been considered, in epidemiological studies on allergy. 

Zhao et al. assessed the frequency of positive APT to Dermatophagoides in a self-selected population in Beijing (healthy university student volunteers). Of the 201 students that were studied, 25.9% had positive results to APT. In subjects with no history of AD, rhinitis, or asthma, the positivity rate of APT was 13.6%; however, it was much lower than that of subjects who had rhinitis or asthma (56.1%, P<0.05). These findings confirmed that APT to Dermatophagoides is highly related not only to AD but also to respiratory allergy (34). 

The most recent survey investigated an unselected pediatric population in Italy. This cross-sectional survey was based on a specific questionnaire, containing the three core ISAAC (International Study on Asthma and Allergy in Children) modules asking about the diagnosis of AD, AR, wheezing, and asthma and the use of SPT and APT. This survey was applied on the entire scholastic population attending a Primary school and a Junior Secondary school in the rural town of San Marco in Lamis, a town of 12,000 inhabitants (Puglia, Italy). Of the 456 questionnaires returned, 279 (61.2 %) were negative for any sign or symptom, while 177 (38.8 %) were positive for symptoms of AD, rhinitis, or asthma/wheezing. In particular, 129 (28.3 %) were positive for only one kind of symptom and 48 (10.5 %) were positive for more than one kind of symptom. Among these subjects, 78 (17.1 %) had a positive SPT and 57 (12.5 %) had a positive ATP. In particular, 13.4 % of subjects were positive only to SPT and 8.8 % were positive only to APT, with dust mites being the allergen that was most frequently positive, while pollen positive results almost exclusively concerned the SPT (35). These findings highlight the importance of the APT also in epidemiological studies, because in an unselected population of children the prevalence of positive results to APT was not so distant from the positive results to SPT, and in 8.8 % of subjects the atopy patch test was the only positive test. Moreover, this confirms the observations from clinical studies that APT may give positive results in concordance with SPT but may also be the only positive test. This would suggest to add the APT in future epidemiological studies on respiratory allergy, especially in the case of AD, to avoid overlooking the non-negligible portion of patients with T-cell-mediated allergy.

## Conclusion

The recent literature provided evidence which redefined the role of the APT in both the diagnosis and epidemiology of AR. Epidemiology is a powerful tool in investigating the importance of allergic diseases but thus far was based on questionnaires addressing the common symptoms of allergy and, quite often, was based on diagnostic tests such as SPT or specific IgE measurement. Even though it is a common fact that the IgE-mediated mechanism drives the majority of allergies, other mechanisms are also important. Thus, the lack of use of the APT is likely to cause an underestimation of the prevalence of allergy, which is of particular importance for AD. In addition, the recent research demonstrated that the APT is frequently positive in patients with respiratory allergy, especially when the patients have a history of current or past AD. The fact that the APT may be the only positive test in patients with respiratory allergy suggests that this test must be included in the diagnostic work-up of AR. Otherwise, patients with negative results to SPT or in vitro IgE tests, and even with negative results to modern diagnostic techniques such as CRD or BAT, may be erroneously classified as nonallergic. Adding the APT allows the physician to achieve a correct diagnosis. 
